# Use of Cold Atmospheric Plasma to Detoxify Hazelnuts from Aflatoxins

**DOI:** 10.3390/toxins8050125

**Published:** 2016-04-26

**Authors:** Ilenia Siciliano, Davide Spadaro, Ambra Prelle, Dario Vallauri, Maria Chiara Cavallero, Angelo Garibaldi, Maria Lodovica Gullino

**Affiliations:** 1 Agroinnova—Centre of Competence for the Innovation in the Agro-Environmental Sector, University of Turin, Largo Paolo Braccini, 2 Grugliasco, Turin 10095, Italy; ilenia.siciliano@unito.it (I.S.); angelo.garibaldi@unito.it (A.G.); marialodovica.gullino@unito.it (M.L.G.); 2 Department of Agricultural, Forest and Food Science (DISAFA), University of Turin, Largo Paolo Braccini, 2 Grugliasco, Turin 10095, Italy; ambra.prelle@gmail.com; 3 Tecnogranda SpA, Via G.B. Conte, 19 Dronero, Cuneo 12025, Italy; dario.vallauri@tecnogranda.it (D.V.); mariachiara.cavallero@tecnogranda.it (M.C.C.)

**Keywords:** aflatoxins, cold atmospheric pressure plasma, detoxification, hazelnut, nitrogen, nuts, temperature, time

## Abstract

Aflatoxins, produced by *Aspergillus flavus* and *A. parasiticus*, can contaminate different foodstuffs, such as nuts. Cold atmospheric pressure plasma has the potential to be used for mycotoxin detoxification. In this study, the operating parameters of cold atmospheric pressure plasma were optimized to reduce the presence of aflatoxins on dehulled hazelnuts. First, the effect of different gases was tested (N_2_, 0.1% O_2_ and 1% O_2_, 21% O_2_), then power (400, 700, 1000, 1150 W) and exposure time (1, 2, 4, and 12 min) were optimized. In preliminary tests on aflatoxin standard solutions, this method allowed to obtain a complete detoxification using a high power for a few minutes. On hazelnuts, in similar conditions (1000 W, 12 min), a reduction in the concentration of total aflatoxins and AFB_1_ of over 70% was obtained. Aflatoxins B_1_ and G_1_ were more sensitive to plasma treatments compared to aflatoxins B_2_ and G_2_, respectively. Under plasma treatment, aflatoxin B_1_ was more sensitive compared to aflatoxin G_1_. At the highest power, and for the longest time, the maximum temperature increment was 28.9 °C. Cold atmospheric plasma has the potential to be a promising method for aflatoxin detoxification on food, because it is effective and it could help to maintain the organoleptic characteristics.

## 1. Introduction

Italy is the second country, after Turkey, for production of hazelnuts with about 85,000 tons in 2012, representing around 9% of global supply (914,000 tons) [1]. Hazelnut (*Corylus avellana* L.) cultivar Tonda Gentile Trilobata is widely cultivated in Piedmont, Northern Italy (15,000 tons in 2014), where it is well adapted to continental climate and it reaches an excellent quality. Confectionery companies process approximately 90% of Italian hazelnut production, while fresh consumption represents the remaining 10%. Hazelnuts are consumed both in-shell and shelled. In-shell hazelnuts are generally used as a snack for fresh consumption, while shelled ones are employed as a raw material for food or cosmetic companies [2].

**Figure 1:**
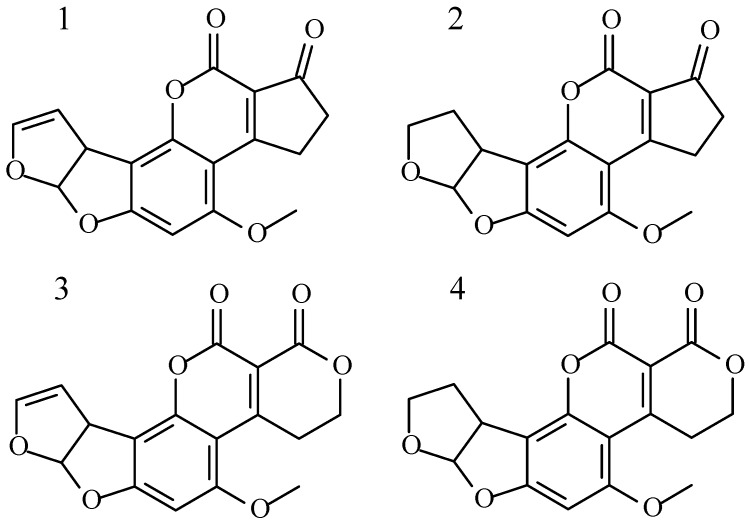
Structures of the four aflatoxins. AFB_1_ (**1**); AFB_2_ (**2**); AFG_1_ (**3**) and AFG_2_ (**4**).

Aflatoxins (AFs) can be found in various foodstuffs, such as nuts, cereals, and spices [3]. Hazelnuts can be contaminated by fungal species belonging to the genus *Aspergillus* that are able to produce AFs. In particular, *A. parasiticus* is able to produce the four main AFs: B_1_ (AFB_1_), B_2_ (AFB_2_), G_1_ (AFG_1_), and G_2_ (AFG_2_), while *A. flavus* is able to produce only AFB_1_ and AFB_2_ (Figure 1). AFs are highly substituted coumarins, AFB_1_ and AFB_2_ have a difuro-coumaro-cyclopentenone structure and emit blue fluorescence, while a six-membered lactone ring replaces the cyclopentenone in AFG_1_ and AFG_2_, which emit yellow-green fluorescence [4].

AFB_1_ and AFG_1_ have also an olefinic double bond at the C_8_–C_9_ position, whereas AFB_2_ and AFG_2_ lack this bond and are less toxic. The four compounds are closely related and the presence of the furocoumarin configuration places them among a large group of naturally occurring compounds with many toxicological activities. Aflatoxins are genotoxic and carcinogenic and can cause both acute and chronic toxicity in humans [5]. AFs were classified by the International Agency for Research on Cancer (IARC) as carcinogenic agents to humans and animals [6]. The presence of furan ring, lactone ring, and C_8_–C_9_ double bond is associated with mutagenicity, carcinogenicity and teratogenicity of aflatoxin B_1_. The destruction of the C_8_–C_9_ double bond on the furan ring reduces the toxicity of aflatoxin B_1_ [7]. AFB_1_-*exo*-8,9-epoxide, catalyzed by cytochrome P450 in mammal liver, is responsible for the carcinogenicity and mutagenicity of AFB_1_ [8]. The serious health and economic consequences of aflatoxin contamination have created the need for legislative limits, rapid detection techniques, and detoxification strategies [9]. In Europe, maximum levels in foodstuffs for AFB_1_, aflatoxin M_1_, and for the sum of aflatoxins (AFB_1_, AFB_2_, AFG_1_ and AFG_2_) in nuts are specified by the Commission Regulation (EU) No 165/2010 [10]. Thresholds for AFB_1_ and total AFs in hazelnuts for direct human consumption and for use as ingredient in foodstuffs are 5 and 10 µg/kg, respectively.

In a batch, nuts contaminated by aflatoxins cannot be visually distinguished from healthy nuts [11]. Aflatoxins tend to accumulate in the external parts of the nuts, particularly in the shell and episperm, where the aflatoxigenic fungi are developing [12]. Detoxification can be very useful in order to recover contaminated commodities. Several detoxification methods, including physical, chemical, or biological tools, were tested against aflatoxins with different degree of success [13].

Plasma, the highly energized fourth state of matter, has been used since the 1990s for bio-decontamination of heat-sensitive materials. The free electrons present in a gas can be accelerated by applying a voltage, to collide with neutral gas atoms causing excitation or ionization. Ionization releases an abundance of highly reactive chemical species, such as positive ions, electrons, excited atoms, UV photons, radicals, and reactive neutral species (reactive oxygen species, ROS, and reactive nitrogen species, RNS) [14]. Depending on the plasma generation system and on the type of gas used, the plasma produced can contain different excited atoms and molecules, ionized gases, radicals, and free electrons [15]. According to the conditions of plasma generation, thermal or non-thermal plasma can be created. The first one is generated at high pressure (≥10^5^ Pa) and with a minimum power of 50 MW. At these conditions the temperatures reached can be very high (from 5 to 20 × 10^3^ K). Non-thermal plasma is obtained at lower pressure and power. Atmospheric pressure techniques, besides being cheaper, have the advantage of allowing the formation of a wide range of active species that can react with the macromolecules of contaminants [16]. In the last decade, dielectric barrier discharge (DBD) plasma was applied to inactivate several microorganisms [17,18], including species of *Aspergillus* and *Penicillium* [19,20].

This technology is based on an electrical discharge that works at atmospheric pressure. The energy required for the discharge is delivered by a power supply through a quasi-alternative voltage with a frequency in the range 100–150 kHz and a maximum power of 2 kW. The reactive species generated in the discharge depend on the energy applied.

The purpose of this study was to understand the efficacy of low temperature atmospheric pressure plasma treatment on the degradation of AFs *in vitro* and on hazelnuts. For this study, a prototype of dielectric barrier discharge (DBD) cold atmospheric plasma system was designed. Different gas mixtures ionized to generate the plasma, different powers applied, and different exposure times were tested to evaluate the efficacy of cold atmospheric plasma on detoxification aflatoxins, both *in vitro* and on raw hazelnuts without shell.

## 2. Results and Discussion

The efficiency of cold atmospheric plasma, generated by ionization of nitrogen and different mixtures of nitrogen/oxygen, was investigated on the reduction of four aflatoxins (AFB_1_, AFG_1_, AFB_2_ and AFG_2_). Three exposure times, ranging from 1 to 4 min, were used at the power of 1000 W. Results showed that an increased presence of O_2_ reduced the efficacy of the cold plasma treatment (Table 1): When oxygen was 21%, the residual AFB_1_ after plasma treatment was 100%, regardless of exposure time. The efficiency of plasma treatment depended on the nature of gases used to form plasma. The highest detoxification efficacy was obtained with nitrogen or nitrogen/oxygen mixtures (0.1% O_2_) for plasma generation and the longest exposure times. The experiments were conducted at atmospheric pressure and in presence of aqueous solutions of the aflatoxins, both factors favoring the production of highly reactive species (OH, O^2−^, H_2_O_2_, O_3_) that can react with the molecules in the solutions [16]. Different studies reported the efficacy of plasma generated with helium or argon for the inactivation of different species of bacteria or fungal spores [21]. Nitrogen has been widely evaluated for plasma generation, as a substitute of the more expensive noble gases, with several publications reporting the efficacy of this type of plasma treatments [22,23,24]. Takamatsu *et al.* [25] investigated the microbial inactivation using non-thermal atmospheric plasma generated with different gases (Ar, O_2_, N_2_, CO_2_, air), and they demonstrated that CO_2_ and N_2_ plasmas inactivated *Escherichia coli*, *Pseudomonas aeruginosa*, and *Staphylococcus aureus* better than other tested gases. The presence of oxygen (O_2_) starts the reaction of atomic oxygen with the nitrogen that generates mainly nitrite and nitrate instead of OH radicals [26].

Based on the results about the effect of the gas used on the reduction of aflatoxins, a second set of experiments was designed by using nitrogen for plasma generation. The parameters modified were the power used and the time of exposure to plasma. Experiments on standard solutions showed that the four aflatoxins were completely eliminated with treatments at 400 W for 12 min (Table 2). An increased treatment effectiveness was observed with increasing the power for plasma generation, from 400 W, to 700 W, 1000 W, and 1150 W (Table 2). A higher power permitted to reduce the treatment times to get a complete aflatoxin removal from aqueous solutions. When the highest power was used, 1 min was sufficient to reach a total detoxification of the four AFs.

Based on the results *in vitro*, a similar scheme of plasma treatments were performed on hazelnuts artificially contaminated with aflatoxins. None of the treatments performed on hazelnuts permitted to obtain a total detoxification, but there was a clear trend towards higher detoxification efficacy with increasing either time or power of treatments, similarly as obtained in the *in vitro* experiments (Table 2). The highest detoxification of AFB_1_ (29.1 ± 5.89) and total AFs (30.4 ± 9.04) was obtained at the highest power (1150 W) with the longest exposure time (12 min). Previously, Basaran *et al.* [27] evaluated the efficacy of cold atmospheric air plasma at 300 W for times ranging from 5 to 20 min against *Aspergillus parasiticus* and the four aflatoxins, showing an average reduction of 51% on AFs. In our experiments, cold atmospheric nitrogen plasma and higher powers of treatment permitted to obtain a similar reduction at 400 W (54.1% residual AFs), but a higher efficacy at 1000 W (25.8% residual AFs). The efficiency of non-thermal plasma is associated with the ability to penetrate into the materials and with the quantity of reactive species formed by nitrogen during plasma treatments [28]. Atomic nitrogen and water generate OH radicals in accordance with the formula: 2N + 2H_2_O → N_2_ + 2OH·+ 2H and they can be responsible for bacterial and fungal inactivation [29]. Dasan *et al.* [30] investigated the effects of atmospheric pressure plasma decontamination on hazelnuts against *A. flavus* and *A. parasiticus* with scanning electron microscopy. They observed that the integrity of cell structure was completely lost after treatment, by causing cell death and loss of aflatoxins production. The efficacy of the treatment could also depend on the distance between electrodes and target.

**Figure 2:**
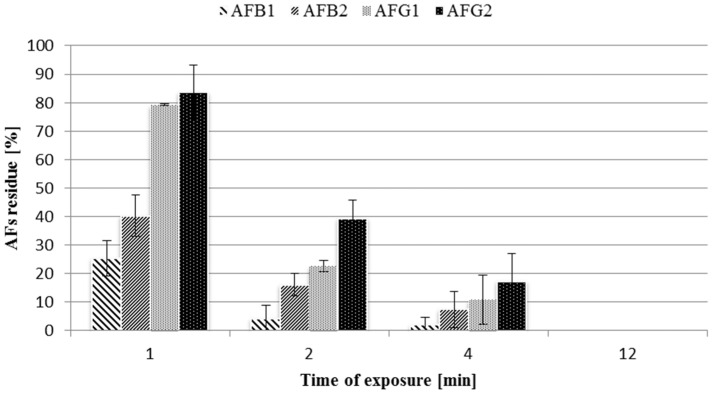
Residue of AFB_1_, AFB_2_, AFG_1_ and AFG_2_ after a treatment at 400 W on standard solutions.

Aflatoxins B_1_ and G_1_ were more sensitive to plasma treatments compared to aflatoxins B_2_ and G_2_, respectively (Figure 2). In addition, Basaran *et al.* [27], using cold atmospheric air plasma on hazelnuts, showed that AFG_2_ was the least sensitive mycotoxin. This is in accordance with the mechanism described by McKenzie *et al.* [31] that suggests a direct ozone attack to the double bonds of AFB_1_ and AFG_1_, while AFB_2_ and AFG_2_ are less reactive to ROS because of the lack of C_8_–C_9_ double bond. This is a very positive feature, because AFB_1_ and AFG_1_ are the most toxic aflatoxins. The process of degradation of aflatoxins using ozonation has been widely studied [32]. The opening of terminal furan ring is promoted by the reaction of ozone with the C_8_–C_9_ double bond that is the most reactive site. OH radicals, that are strong oxidizing agents, increase the ability of ozone to react with the olefinic site of AFB_1_ [33].

Degradation of AFB_1_ has been widely studied [34,35]. The detoxification of AFB_1_ initially involves the formation of a β-keto acid structure due to the opening of the lactone ring followed by the formation of aflatoxin D_1_ or aflatoxin D_2_ [36]. Additionally, AFG_1_ degradation has been investigated by Velazhahan *et al.* [37], who suggest the modification of lactone ring structure with the formation of two different metabolites. Cytochrome P450 catalyzes the formation of the epoxide group, considered the carcinogenic and mutagenic form of AFB_1_. After the opening of lactone ring and the loss of the C_8_–C_9_ double bond, the formation of AFB_1_-*exo*-8,9-epoxide is blocked [38]. As reported by McKenzie *et al.* [31], ozonolysis induce AFB_1_ degradation and the breakdown products are non-toxic or lower toxic compounds, due to complete degradation of toxins or partly chemical modification. The identity and toxicity of breakdown products of aflatoxins after plasma treatment were not determined in this work.

Under plasma treatment, AFB_1_ was more reactive compared to aflatoxin G_1_, in accordance with Baertschi *et al.* [39] that investigated the reactivity of AFB_1_, AFG_1_ and sterigmatocystin in presence of epoxides. Computation studies clearly demonstrated that an electron deficiency on the carbonylic carbon on lactone ring favored the nucleophilic attack that induced the hydrolysis of this site [37].

**Figure 3:**
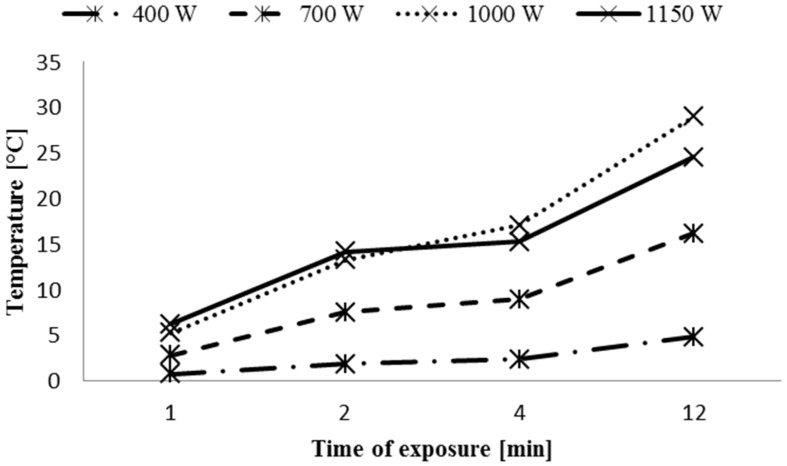
Increase in temperature (∆T °C) during treatments.

Temperature in the treatment zone was monitored throughout the experiments at the beginning and at the end of treatment. In Figure 3, the increase in temperature during plasma treatments at different powers are reported.

Higher powers led to an increase of ∆T, with the maximum increment, equal to 28.9 °C (T_max_ = 58.9 °C), reached using the longest time exposure at the maximum power. These ∆T are not expected to affect the qualitative properties of hazelnuts, making the plasma treatment compatible with the food processing. On the contrary, the use of atmospheric microwave-induced argon plasma to degrade three mycotoxins (AFB_1_, nivalenol and deoxynivalenol) resulted in temperature increases over 100 °C [13]. In the choice of the treatment, we should consider that microwave-induced argon plasma is quicker, because the treatment lasts a few seconds, while cold atmospheric plasma is effective after a few minutes of treatment, but this latter one could help to maintain the organoleptic characteristics.

## 3. Conclusions

Cold atmospheric pressure plasma is quite well established in various industrial processes, and its use is also potentially promising in the food sector, in particular for microbial and mycotoxin inactivation [29,30]. The possibility to modify the operating conditions, such as power, gas composition, and time of treatment, permits to adapt this technology to naturally contaminated food matrices. In the scaling up of the prototype, the system could be included in the hazelnut processing chain, after dehulling and before roasting. Compared to microwave-induced argon plasma, this treatment lasts longer, but it looks more promising because it does not increase significantly the temperature of the food matrix. Further, naturally contaminated hazelnuts and the different parts of nuts, such as shell, episperm, and kernel will be analyzed. By considering the effect of the treatment on aflatoxins, the amount and toxicity of the breakdown products will be determined. Future works will also evaluate the physical-chemical properties, the structural changes, and the organoleptic properties of the food matrix after treatment.

## 4. Materials and Methods

### 4.1. Materials

AFB_1_ (purity ≥ 98%), AFB_2_ (purity ≥ 98%), AFG_1_ (purity ≥ 98%) and AFG_2_ (purity ≥ 98%) standards were purchased from Sigma-Aldrich (St. Louis, MO, USA) and dissolved in methanol in order to prepare a working solution of 10 mg/mL. LC-MS grade methanol, acetonitrile acetic acid, and water, used as mobile phases and as extraction solvents, were purchased from Sigma-Aldrich. NaCl, KCl, Na_2_HPO_4_, and KH_2_PO_4_ used to prepare phosphate-buffered saline (PBS) solution were purchased from Merck (Darmstadt, Germany) and dissolved in ultrapure water (Maina, Turin, Italy). AflaTest WB immunoaffinity columns were purchased from Vicam (Watertown, MA, USA).

### 4.2. Atmospheric Pressure Plasma System

**Figure 4:**
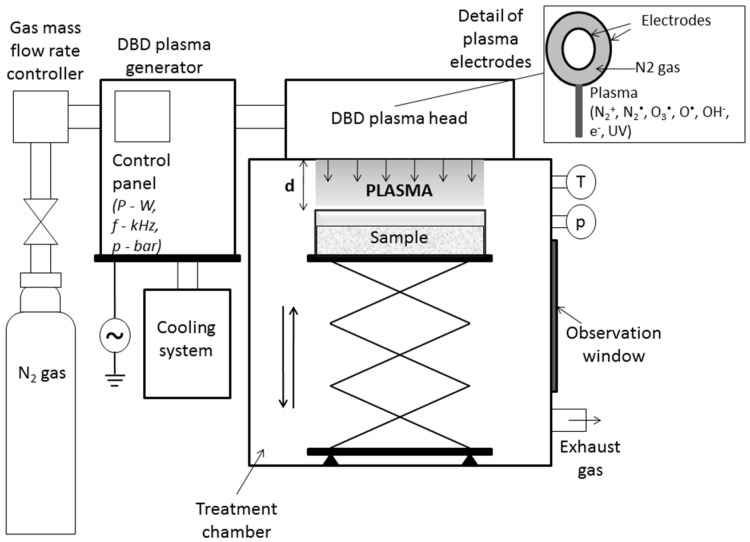
Schematic diagram of the plasma system used in this work for treatments.

A dielectric barrier discharge (DBD) cold atmospheric plasma system produced by AcXys Technologies (St. Martin Le Vinoux, France) was used (Figure 4). The system was modified to include an isolated chamber in order to maintain the sample under controlled atmosphere and temperature. The plasma technology is developed around an electrical discharge that works at an operative pressure of about 7 bar. The central electrode is covered with a dielectric coating to prevent any cathode spot that could derive in arcs. The gap between the electrodes is 1 mm. The outer electrode is made out of aluminum steel. Gas mixture flows through the structure crosswise. It travels in and out through two slots longitudinally opposed. Flow velocity and gas mixture can be controlled according to the process requirement. The DBD plasma system can work with pure N_2_ gas or mixtures with a prevalence of N_2_, including air. The standard operative gas flow was about 120 L/min. The energy fed to the discharge was delivered by a power supply through a quasi-alternative voltage with a frequency in the range 100–150 kHz and a power in the range between 0.4 and 2 kW. Cooling was added to keep the system under safe mode of operation. The parameters that could be controlled are electrical power, time of exposure, gas composition, and distance of the treated samples from the plasma source. In all the experiments, the distance was fixed at about 50 mm.

### 4.3. Efficacy of Gas Mixtures on Aflatoxin Detoxification

A mixture of AFs standards were prepared by diluting original standards at the final concentration of 10 ng/mL in Petri dishes containing 10 mL of water. Pure N_2_, and three mixtures of nitrogen/oxygen (21%, 1%, and 0.1% of O_2_) were used for plasma generation. The samples were treated with a power of 1000 W for three exposure times (1, 2, and 4 min). Each treatment was performed on three sample replicates. The experiments were performed twice.

### 4.4. Effect of Power and Exposure Time on Aflatoxin Detoxification in Vitro

For the second experimental set up, pure N_2_ was chosen for plasma generation. The mixture of AFs standards were prepared as previously described. The samples were treated with four powers (400 W, 700 W, 1000 W, 1150 W) applied for four exposure times (1, 2, 4, and 12 min). Each treatment was performed on three sample replicates. The experiments were performed twice.

### 4.5. Effect of Power and Exposure Time on Aflatoxin Detoxification in Hazelnut

Raw hazelnuts free from aflatoxins were harvested in Cortemilia (44°34′44′′04 N, 08°11′38′′40 E), Northern Italy, and provided by La Gentile s.r.l. Raw hazelnuts without shell were artificially contaminated at the final concentration on hazelnuts of 20 ng/g each aflatoxin by spraying a solution of the four AFs. For each treatment, 40 g of contaminated hazelnuts were used. The samples were treated with four powers (400 W, 700 W, 1000 W, 1150 W) applied for four exposure times (1, 2, 4, and 12 min). Each treatment was performed on three sample replicates. The experiments were performed twice. Throughout the experiments, the temperature of the chamber was monitored through a temperature probe placed at about 2 cm from the plasma source.

### 4.6. Extraction, Clean-up and LC-MS/MS Conditions

The extraction method described by Prelle *et al.* [40] was used. Twenty-five grams of grounded hazelnuts were extracted with 5 g of NaCl and 125 mL of a H_2_O:CH_3_OH (20:80 *v*/*v*) solution for 2 h on a rotary shaker. The extract was filtered through Whatman No.4 filter paper to eliminate solid particles and then filtered by using a Whatman PDVF 0.45 µm syringe filter (Whatman GmbH, Dussel, Germany). An aliquot of 10 mL of filtrate was diluted 1:1 in ultrapure water and used for purification procedure using immunoaffinity columns. After conditioning, diluted samples were loaded and flushed under a flow of 0.5 mL/min. Columns were washed with PBS solution and water. AFs were eluted with 3 mL of methanol and the eluate was evaporated to dryness. One mL H_2_O:CH_3_OH (90:10 *v*/*v*) acidified with 0.1% acetic acid was added to the residue and vortexed before HPLC-MS/MS analysis.

Analysis was performed using a previously validated method [40] using Varian Model 212-LC micro pumps (Palo Alto, CA, USA) with a Varian autosampler Model 410 Prostar coupled with a Varian 310-MS triple quadrupole mass spectrometer with an electrospray ion source operating in positive ionization mode. For chromatographic separation, a Pursuit XRs Ultra C18 (100 mm × 2.0 mm, 2.8 µm, Varian, Cernusco sul Naviglio (MI), Italy) analytical column was used. Column temperature was set at 30 °C, eluents were H_2_O (A) and CH_3_OH (B), both acidified with 0.1% CH_3_COOH, flow was set at 0.2 mL/min. The gradient was set as follows: 0–25 min from 90% to 15% of A; 25–28 min from 15% to 90% of A; 28–30 min 90% of A. Monitoring reaction mode (MRM) transitions used for quantification were: 313 > 285 (CE 14 V) for AFB_1_, 315 > 287 (CE 18 V) for AFB_2_, 329 > 243 (CE 18 V) for AFG_1_, 331 > 245 (CE 24 V) for AFG_2_. The collision gas (Ar) pressure was set at 2 mbar for all experiments.

AFs quantification was performed using external calibration based on serial dilution of a multi-analyte stock solution. Results were corrected by recoveries that had been determined by spiking five different blank samples at three concentration levels.

## Figures and Tables

**Table 1:** Residual (%) aflatoxin B_1_ and total aflatoxins on aqueous standard solutions after treatments with atmospheric plasma generated with different gas mixtures and applied for different exposure times (Power = 1000 W).

Plasma	AFB_1_ (%)			Afs ^1^ (%)		
gas	1 min	2 min	4 min	1 min	2 min	4 min
21% O_2_	100	100	100	99.44	95.50	94.9
1% O_2_	100	50.2 ± 17.1	0.50 ± 0.29	100	88.5 ± 7.55	50.5 ± 14.0
0.1% O_2_	78.1 ± 0.07	31.3 ± 5.98	9.31 ± 0.27	92.3 ± 2.89	73.8 ± 8.49	59.7 ± 1.49
N_2_	87.5 ± 17.4	20.0 ± 0.69	0	77.9 ± 11.1	32.8 ± 4.72	13.7 ± 0.19

^1^ average residual concentration of AFB_1_, AFB_2_, AFG_1_, and AFG_2_, after plasma treatment.

**Table 2:** Residual (%) aflatoxin B_1_ and total aflatoxins on aqueous standard solutions and on contaminated hazelnuts after treatments with atmospheric plasma generated with different powers and applied for different exposure times.

Power (W)	Time (min)	Standards		Hazelnuts ^1^	
		AFB_1_ (%)	AFs ^2^ (%)	AFB_1_ (%)	AFs ^2^ (%)
400	1	25.4 ± 6.13	57.1 ± 28.8	100 ± 1.60	97.7 ± 13.6
	2	7.75 ± 0.17	20.6 ± 14.6	100 ± 5.10	98.7 ± 23.9
	4	4.49 ± 0.80	9.40 ± 6.41	83.2 ± 27.5	81.2 ± 30.0
	12	0	0	54.3 ± 0.91	54.1 ± 6.01
700	1	12.7 ± 6.08	44.3 ± 25.6	99.6 ± 68.6	90.9 ± 41.6
	2	0	6.61 ± 1.94	84.7 ± 15.2	84.7 ± 10.2
	4	0	2.40	83.1 ± 6.81	83.9 ± 30.2
	12	0	0	44.8 ± 3.36	52.9 ± 10.7
1000	1	9.52 ± 4.70	24.0 ± 12.1	96.2 ± 6.50	88.5 ± 12.9
	2	0	0.60	82.5 ± 30.2	79.9 ± 34.8
	4	0	0	78.5 ± 10.7	66.9 ± 11.8
	12	0	0	35.7 ± 0.87	25.8 ± 13.2
1150	1	0	0	85.2 ± 8.46	81.7 ± 20.3
	2	0	0	60.2 ± 3.31	62.0 ± 13.4
	4	−	−	50.6 ± 9.77	60.0 ± 20.8
	12	−	−	29.1 ± 5.89	30.4 ± 9.04

^1^ Mean Initial concentrations of AFB1, and AFs where 8.02 ng/g and 36.11 ng/g respectively; ^2^ Average residual concentration of AFB_1_, AFB_2_, AFG_1_, and AFG_2_, after plasma treatment.
